# Prediction of obstructive coronary artery disease in patients undergoing heart valve surgery: A cross-sectional study in a tertiary care hospital

**DOI:** 10.34172/jcvtr.2023.30557

**Published:** 2023-03-16

**Authors:** Sy Van Hoang, Hai Phuong Nguyen Tran, Kha Minh Nguyen, Phong Thanh Tran, Khoa Le Anh Huynh, Nghia Thuong Nguyen

**Affiliations:** ^1^Department of Internal Medicine, Faculty of Medicine, University of Medicine and Pharmacy at Ho Chi Minh City, Ho Chi Minh City 700000, Vietnam; ^2^Department of Cardiology, Cho Ray Hospital, Ho Chi Minh City 700000, Vietnam; ^3^Department of Cardiology Intervention, Cho Ray Hospital, Ho Chi Minh City 700000, Vietnam; ^4^Department of Cardiology, Can Tho Central General Hospital, Can Tho City 900000, Vietnam; ^5^Department of Biostatistics, Virginia Commonwealth University School of Medicine, Virginia, USA

**Keywords:** Obstructive Coronary Artery Disease, Valvular Heart Surgery, Support Vector Machine, Logistic Regression, Decision Tree

## Abstract

***Introduction:*** Estimating the probability of obstructive coronary artery disease in patients undergoing noncoronary cardiac surgery should be considered compulsory. Our study sought to evaluate the prevalence of obstructive coronary artery disease in patients undergoing valvular heart surgery and to utilize predictive methodology of concomitant obstructive coronary artery disease in these patients.

***Methods:*** The retrospective study cohort was derived from a tertiary care hospital registry of patients undergoing coronary angiogram prior to valvular heart operations. Decision tree, logistic regression, and support vector machine models were built to predict the probability of the appearance of obstructive coronary artery disease. A total of 367 patients from 2016 to 2019 were analyzed.

***Results***: The mean age of the study population was 57.3±9.3 years, 45.2% of the patients were male. Of 367 patients, 76 (21%) patients had obstructive coronary artery disease. The decision tree, logistics regression, and support vector machine models had an area under the curve of 72% (95% CI: 62% - 81%), 67% (95% CI: 56% - 77%), and 78% (95% CI: 68% - 87%), respectively. Multivariate analysis indicated that hypertension (OR 1.98; *P*=0.032), diabetes (OR 2.32; *P*=0.040), age (OR 1.05; *P*=0.006), and typical angina (OR 5.46; *P*<0.001) had significant role in predicting the presence of obstructive coronary artery disease.

***Conclusion:*** Our study revealed that approximately one-fifth of patients who underwent valvular heart surgery had concomitant obstructive coronary artery disease. The support vector machine model showed the highest accuracy compared to the other model.

## Introduction

 Valvular heart disease is a condition that affects the valves of the heart, which can lead to a range of complications such as heart failure and stroke. The underlying causes of valvular heart disease can be diverse and may vary depending on the region and socioeconomic status of the population. In middle-income countries, where access to healthcare and resources may be limited, there may be different strategies for approaching the diagnosis and management of valvular heart disease compared to high-income countries with more advanced healthcare systems. This may include the use of simpler diagnostic tests and less invasive treatment options in middle-income countries, while high-income countries may have access to more advanced diagnostic tools and surgical interventions. The rheumatic and degenerative etiology was still considered the most common cause of valvular heart disease. In developing countries, including Vietnam, rheumatic heart disease remains the leading cause of valve heart disease.^1^ These valve lesions were often observed in young people and had a very high risk of atherosclerosis. The degenerative or atherosclerotic cause was the most common etiology of valvular heart disease in developed countries.^2^ Therefore, the prevalence of coronary artery disease (CAD) is lower. Furthermore, with increased life expectancy, rheumatic heart disease has been replaced by degenerative heart disease in the world.^1^ In parallel, the prevalence of obstructive CAD was also increasing. Depending on the type of valve injury, the representation of obstructive CAD also varies.^3,4^

 In clinical practice, obstructive coronary artery disease was defined in patients undergoing heart valve surgery that would greatly affect the success of the operation and long-term outcomes in these patients. In the literature, concomitant obstructive coronary heart disease in patients with valvular heart surgery affects the success of the operation and long-term results. Therefore, correctly diagnosing obstructive CAD is crucial.^5^ According to the European Society of Cardiology guideline, preoperative coronary angiography was performed in men over 40 years of age or women postmenopause, and noninvasive functional tests could not confirm obstructive CAD. In the ESC guideline, coronary computed tomography angiography plays a vital role in patients with a low probability of obstructive coronary artery disease (class of recommendation IIa, level of evidence C) due to its high negative predictive value.^6^ The determination of obstructive CAD based on emerging developed countries guidelines does not seem to be the best approach in other countries in the world. Coronary angiography was considered an invasive procedure without free complications, and it was a high-cost procedure in low- to middle-income countries.^4^ As applied in the general population, the development of models to evaluate the pre-test probability of obstructive CAD is necessary to prefer patients suitable for different preoperative strategies, therefore reducing invasive and unnecessary procedures in patients with a lower pre-test probability of obstructive CAD. The purpose of this study is to evaluate the proportion of obstructive CAD in patients undergoing valve surgery and to develop a stool that predicts obstructive coronary artery disease.

## Materials and Methods

###  Study population

 The study population of 367 patients, who underwent cardiac surgery in the Department of Cardiology Surgery at Cho Ray Hospital, Ho Chi Minh City, Vietnam, between 2016 and 2019, was retrospectively recorded. Clinical variables and biochemical parameters were evaluated. The coronary angiogram was performed before the operation.

 Exclusive criteria of the study included patients who had known CAD, or patients who have been diagnosed with congenital cardiac diseases, or patients with a history of acute myocardial infarction for three months ago, or patients under 18 years of age, or those with a previous history of operation or repair related to cardiac valves, or patients who are the medical report lack the necessary information.

 The presence of obstructive coronary artery disease was defined as 50% stenosis of at least the main coronary arteries as evaluated by the interventional cardiologist performing the procedure. In addition, traditional cardiac risk factors such as age, sex, hypertension, diabetes, chronic kidney disease, and a family history of premature cardiovascular disease were reviewed. Cardiovascular diseases such as hypertension, diabetes, hyperlipidemia, and chronic kidney disease were identified by ICD-10 coding. Biochemical parameters such as liver transaminase levels, lipid panel, kidney function, and HbA1C were recorded. Types of valve pathology and echocardiographic parameters, including left ventricular ejection fraction and PAPs, were collected according to the results of transthoracic echocardiography.

###  Preparation data

 Given an originally imbalanced data set (21% obstructive CAD), we utilized a random sampling strategy to increase the proportion of positive cases in the dataset. We aim to improve the sensitivity (better to classify positive cases). The improvement would help algorithms to generate models that had better predictions. However, there was a trade-off between sensitivity and specificity, in such a way that if we increase sensitivity, the specificity would decrease, and vice versa.^7,8^ Because of that, we need to balance positive obstructive CAD and negative obstructive CAD. We randomly divided our data into train datasets (60%) and tests (40%). Then, both sampling methods would be utilized in the training dataset. In both sampling methods, we would duplicate both the positive and negative cases, but the number of entry duplication for the positive case will be a much higher rate. Our last train data consisted of 52% for non-obstructive CAD and 48% for obstructive CAD.

## Machine learning model

###  Logistics regression


(1) $$p\left(obstructive=1\left|X\right.\right)=\frac{1}{1+e^{-\beta^{T}X}}$$

The probability that patients had obstructive CAD, given that the predictors followed equation (1), and the estimate coefficient β presented in Table 1. We would classify the new patients A having obstructive CAD as yes if the probability was greater than 0.5. For example, if a new patient’s characteristics followed: premature cardiovascular disease = no, hypertension = yes, smoking = yes, diabetes = no, chronic kidney disease = no, lipid disorders = no, age = 64, sex = male, BMI = 26.7, glucose = 71, ALT = 53, AST = 72, BUN = 16, creatinine = 1.21, eGFR = 50.3, LVDd = 47, EF = 69, PAPs = 40, aortic valve = no, mitral valve = no, typical angina = no, and atypical angina = no. The probability of patient having obstructive CAD was 93.6%, and our model classifies patient having obstructive CAD.

 Decision tree: machine learning algorithm that is used for both classification and regression analysis. It is a type of supervised learning algorithm that is based on a tree-like model of decisions and their possible consequences. We will utilize a method to identify the top important features that are related to the outcome.

**Table 1:** Estimate the logistic regression coefficient

**Predictor (X)**	**Coefficient (β)**	**Standard error**	***P*** ** value **
Intercept	-19.94	2.90	< 0.0001
Premature cardiovascular disease	-15.30	319.82	0.9618
Hypertension	1.15	0.13	< 0.0001
Diabetes	1.78	0.20	< 0.0001
Smoking	2.44	0.30	< 0.0001
Chronic Kidney Disease	0.88	0.30	0.0031
Lipid disorders	-0.90	0.18	< 0.0001
Age	0.15	0.01	< 0.0001
Sex	0.16	0.24	0.5075
BMI	0.22	0.03	< 0.0001
Glucose	-0.01	0.00	0.0001
ALT	0.03	0.00	< 0.0001
AST	0.00	0.00	0.6642
BUN	-0.06	0.01	< 0.0001
Creatinine	4.03	0.99	< 0.0001
eGFR	0.09	0.01	< 0.0001
LVEDd	-0.08	0.01	< 0.0001
LVEF	-0.05	0.01	< 0.0001
PAPs	0.03	0.00	< 0.0001
Aortic valve	-0.16	0.25	0.5340
Mitral valve	0.77	0.23	0.0008
Typical Angina	2.82	0.14	< 0.0001
Atypical Angina	-0.04	0.27	0.8837

Abbreviations: BMI, body mass index; ALT, alanine aminotransferase; AST, aspartate aminotransferase; eGFR, estimated glomerular filtration rate; LVEDd, left ventricular end-diastolic diameter; LVEF, left ventricular ejection fraction; PAPs, systolic pulmonary artery pressure

 Support Vector Machine (SVM) is a type of machine learning algorithm used for regression analysis. The basic idea of SVM is to find a hyperplane that maximally separates the classes in the feature space. The algorithm searches for a decision boundary that maximizes the margin, which is the distance between the boundary and the nearest data points of each class.

###  Statistical analysis

 All analyzes were performed using RStudio (V3.6.2, Integrated Development for *R. RStudio*, PBC, Boston, MA). The results were presented as mean ± standard deviation for variables with normality and median with interquartile range (IQR) for variables without normality. The t-test of equal variance was used to compare the two groups. We performed Fisher’s exact test for categorical variables if expected value is less than six and Pearson’s chi-squared test for other categorical variables. Three machine learning algorithms (Support-Vector Machine - SVM, Decision Tree, DT, and Logistics Regression, LR) were performed correctly to classify the positive and negative cases. The accuracy sensitivity, specificity, and area under the curve would be calculated based on each algorithm’s test data to compare which model would have better predictive power: the higher value, the better model.

## Results

 A total of 367 patients underwent cardiac surgery from 2016 to 2019 in Vietnam. The study included 76 patients (21%) who had obstructive CAD. One hundred and sixty-six patients had an aortic valve, 164 patients had a mitral valve, and 37 patients had combined two valves. The mean age of the study population was 57.3 ± 9.3 years. In addition, our data had 166 patients who were male and 201 patients were female. Of these male patients, 156 patients were aged > 40 years, of which 53.9% were obstructive CAD. For female patients, 172 patients were aged > 50 years old, of which 43.4% were obstructive CAD. Furthermore, the mean age of obstructive CAD was 61 ± 8.5 compared to 56 ± 9.2 for patients without obstructive CAD, in addition to 64.5% of patients with obstructive CAD who present hypertension. The difference between the obstructive CAD and non-obstructive CAD groups was significant with hypertension, diabetes, chronic kidney diseases, age, male patients aged 40 years and older, and LVEF. The demographic characteristics of the patients are presented in Table 2.

**Table 2:** Comparison of baseline characteristics between 2 groups

**Characteristics**	**Cohort** **n=367**	**Obstructive CAD**		***P *** **value**
		**No** **n=291**	**Yes** **n=76**	
Age, years	57.3 ± 9.3	56 ± 9.2	61 ± 8.5	< 0.001
Family history of premature cardiovascular disease, n (%)	2 (0.5)	2 (0.7)	0 (0)	NA
Hypertension, n (%)	157 (42.8)	108 (37.1)	49 (64.5)	< 0.001
Diabetes, n (%)	41 (11.1)	22 (7.6)	19 (25.0)	< 0.001
Smoking, n (%)	14 (3.8)	9 (3.1)	5 (6.6)	0.18
Chronic Kidney Disease, n (%)	19 (5.2)	10 (3.4)	9 (11.8)	0.006
Lipid disorders, n (%)	123 (33.5)	97 (33.3)	26 (34.2)	0.99
BMI, kg/m^2^	22.4 ± 3.1	22.3 ± 3.2	22.7 ± 3.0	0.29
Male > 40, n (%)	156 (42.5)	115 (39.5)	41 (53.9)	0.10
Female > 50, n (%)	172 (46.9)	139 (47.8)	33 (43.4)	0.52
Glucose, mg/dL	112.8 ± 43.9	110.4 ± 40.4	121.9 ± 54.9	0.10
ALT, U/L	48.1 ± 33.4	33.5 ± 50.6	33.2 ± 37.4	0.95
AST, U/L	41.8 ± 44.5	41.2 ± 44.7	43.9 ± 43.9	0.63
BUN, mg/dL	17.43 ± 7.1	17.1 ± 6.2	18.7 ± 9.6	0.18
Creatinine, mg/dL	1.2 ± 0.25	1.14 ± 0.23	1.2 ± 0.3	0.10
TG, mg/dL	140.5 ± 102.2	138.7 ± 38.7	142.1 ± 137.2	0.93
HDL-C, mg/dL	37.1 ± 11.8	36.1 ± 9.4	38.1 ± 14.0	0.69
LDL-C, mg/dL	110.1 ± 33.8	117.8 ± 36.4	103.5 ± 31.4	0.32
HbA1C, %	8.5 ± 4.6	7.5 ± 1.8	9.5 ± 6.3	0.35
LVEDd, mm	51.6 ± 10.4	51.5 ± 10.4	52.1 ± 10.2	0.63
LVEF, %	62.8 ± 11.5	63.57 ± 11.19	59.61 ± 12.1	0.01
PAPs, mm Hg	42.2 ± 17.1	42 ± 17.2	43 ± 17	0.63
Aortic valve, n (%)	166 (45.2)	130 (44.6)	36 (47.4)	0.77
Mitral valve, n (%)	164 (44.7)	132 (45.3)	32 (42.1)	0.70

Abbreviations: BMI, body mass index; ALT, alanine aminotransferase; AST, aspartate aminotransferase; BUN, blood urea nitrogen; HDL-C, high density lipoprotein cholesterol; LDL-C, low density lipoprotein cholesterol, LVEDd, left ventricular end-diastolic diameter; LVEF, left ventricular ejection fraction; PAPs, systolic pulmonary artery pressure Values are mean ± standard deviation or frequency (percentage).

###  Decision Tree Model

**Figure 1:**
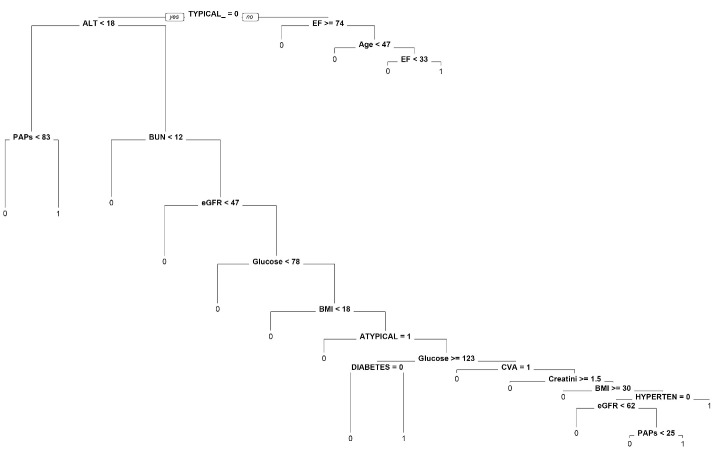
Flowchart patient characteristics for decision tree

 The decision tree directly classified patients for obstructive CAD from the logistic regression model. In addition, it ranks important variables to minimize the path that led to the decision. For example, in Figure 1, if the characteristics of the patients: typical angina = no, ALT < 18, and PAPs < 83, our decision tree would return patients who did not have obstructive CAD. If we consider patient A from logistics regression with CVA = 0, patient A would also classify obstructive CAD = 1.

###  Support vector machine

**Figure 2:**
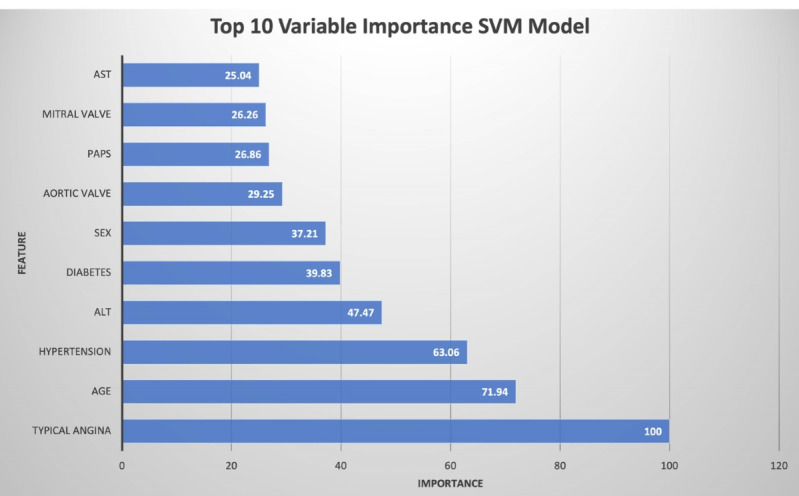
Top 10 Important Variables from the Support Vector Machine Model

 The top 10 crucial variables that improved our SVM were age, PAPs, mitral valve, aortic valve, typical angina, hypertension, ALT, AST, sex, and diabetes (Figure 2). Furthermore, support vector machines also classified patients A as having obstructive CAD. The support vector machine is the linear kernel with an optimized cost of 18 to classify obstructive CAD patients.

###  Predictive comparison

**Figure 3:**
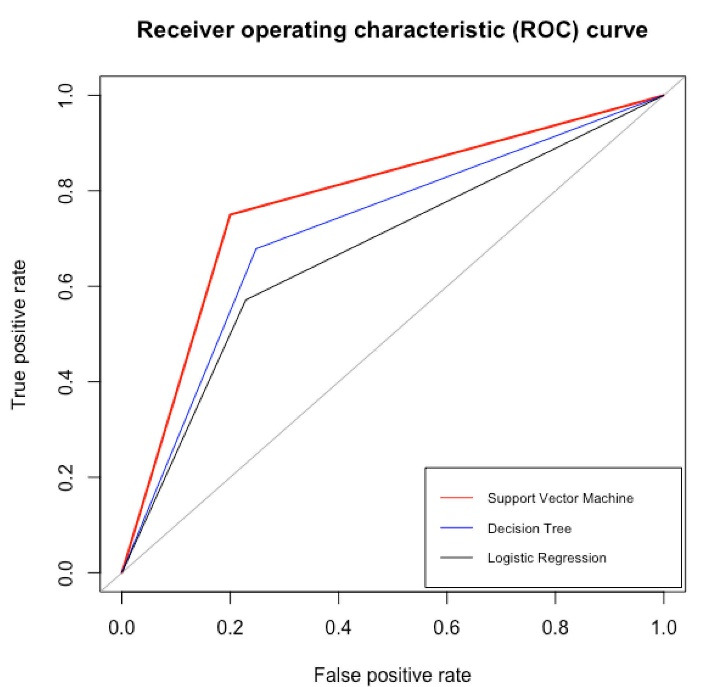
Comparison between the ROC of logistics regression (AUC = 67%), support vector machine (AUC = 78%) and decision tree (AUC = 72%)

 The support vector machine gave the highest precision (79%) compared to logistics regression (73%) and decision tree (74%) on the test data. Furthermore, the model had a higher score on sensitivity (75%) and specificity (80%) compared to logistics regression (sensitivity: 57%, specificity: 77%) and decision tree (sensitivity: 68%, specificity: 75%). Thus, a support vector machine would better classify new patients with obstructive CAD. Furthermore, the area under the curve score for the support vector machine was 0.78 with 95% CI: 0.69 – 0.87 compared to the decision tree was 0.72 with 95% CI: 0.62 – 0.81 and logistic regression was 0.67 with 95% CI: 0.56 – 0.77. The receiver operating characteristic curve is shown in Figure 3.

####  Association between obstructive CAD and significant predictors

 From the univariate analyzes Table 3, the critical predictor variables of the SVM model, such as hypertension, diabetes, typical angina, and age, were associated with increased odds of obstructive CAD. Furthermore, individuals with typical angina had the highest probability of exposure to obstructive CAD (7.57 times) than those without typical angina. Of these predictor variables, the mitral valve or the male sex decreases the risk of obstructive CAD with an odds ratio of 0.88 and 0.60. In the multivariate analyzes presented in Table 4, we had the same statistically significant variable as in the univariate analyzes. Based on multivariate analyzes, typical angina continued to have the most robust relationship with obstructive CAD. Individual patients with typical angina had 5.46 times the risk of obstructive coronary artery disease than patients without typical angina. ALT was one of the top 5 importance variables from SVM; however, we did not find any statistically significant data from univariate and multivariate analyzes.

**Table 3:** Univariate predictors for the top 10 importance variable of the support vector machine model

**Predictors**	**OR (95% CI)**	***P*** ** value**
Hypertension	3.08 (1.82 - 5.21)	< 0.001
Diabetes	4.08 (2.07 - 8.02)	< 0.001
Mitral valve	0.88 (0.53 - 1.46)	0.610
Aortic valve	1.11 (0.67 - 1.85)	0.667
Age	1.08 (1.04 - 1.11)	< 0.001
Male Sex	0.60 (0.36 - 1.00)	0.050
AST	1.00 (1.00 - 1.01)	0.639
PAPs	1.00 (0.99 - 1.02)	0.627
ALT	1.00 (0.99 - 1.01)	0.964
Typical angina	7.57 (4.31 - 13.29)	< 0.001

Abbreviations: AST, aspartate aminotransferase; PAPs, systolic pulmonary artery pressure; ALT, alanine aminotransferase

**Table 4:** Multivariate predictors for the top 10 importance variables from the support vector machine model

**Predictors**	**OR (95% CI)**	***P *** **value**
Hypertension	1.98 (1.06 - 3.68)	0.032
Diabetes	2.32 (1.04 - 5.15)	0.040
Age	1.05 (1.01 - 1.09)	0.006
Male sex	0.56 (0.31 - 1.02)	0.057
ALT	0.99 (0.98 - 1.01)	0.207
AST	1.01 (0.99 - 1.02)	0.418
PAPs	1.01 (0.99 - 1.03)	0.446
Aortic valve	0.66 (0.22 - 1.98)	0.461
Mitral valve	0.90 (0.34 - 2.42)	0.838
Typical angina	5.46 (2.94 - 10.14)	< 0.001

Abbreviations: AST, aspartate aminotransferase; PAPs, systolic pulmonary artery pressure; ALT, alanine aminotransferase

## Discussion

 In our study, the prevalence of obstructive CAD was 21%, consistent with studies in developing countries,^3,9,10^ and lower than that of populations in developed countries.^11,12^ Age has been considered an essential factor in predicting the presence of obstructive CAD. Cazelliet al^9^ revealed a prevalence of 20% in a study of the Brazilian population of 712 patients with a mean age of 58 years. In the literature, the older the population study, the higher the prevalence of obstructive CAD. The mean age was 57.3 years in our study, and Kruczan et al^13^ reported a total prevalence of obstructive CAD of 15.9%, but that of the population cohort with age < 50 years was 6%. Lappéet al^14^ had shown that the observed prevalence of obstructive CAD in American patients undergoing heart valve surgery was 19.3% and the mean age in this survey was 63 years. This author performed in a developed country that showed that the prevalence of obstructive CAD was comparable to our results. This result could be explained by the cohort study conducted between 2004 and 2013 and whose patients with angina symptoms had been excluded. Additionally, in our study, 367 patients underwent cardiac surgery from 2016 to 2019. The prevalence of obstructive CAD had increased in Vietnam due to several risk factors for cardiovascular diseases that were becoming more popular in developing countries.^15^ Our results have proven that rheumatic valvular heart diseases have been replaced by degenerative valvular heart diseases, even in developing countries, including Vietnam.^16^ Although little study had been done in low- to middle-income countries with a large population-based study. To our knowledge, this was the first report on the presence of obstructive CAD in patients undergoing heart valve surgery and provides a predictive model of obstructive CAD in the Southeast Asia area.

 In clinical practice, the 2019 ESC guideline for the management of chronic coronary syndrome of the European Society of Cardiology recommended that the probability of obstructive coronary artery disease assessment was based on age, sex, and characteristics of the probability of obstructive coronary artery disease angina symptoms. If obstructive CAD could not be determined by clinical evaluation alone, non-invasive imaging tools such as coronary computed tomography angiography are recommended as the first-line subclinical test.^17^ Currently, coronary angiography is recommended as the gold standard for diagnosing obstructive coronary artery disease in patients with valvular heart surgery. Although this invasive procedure has been performed widely around the world, it is not free of complications. Then some complications can be encountered, such as vascular injury, hematoma.^18^ In patients with valvular heart surgery, a potential predictive model of obstructive CAD was necessary in the preoperative period. Instead of performing routine coronary angiography, the valuable model would help these patients avoid invasive coronary angiography procedures and save treatment costs. Several models were developed to predict the presence of obstructive coronary disease in patients who underwent valvular heart disease, such as Thalji et al^19^ with C-index = 0.74. However, in addition to using logistics regression, we introduced a support vector machine and a decision tree.

 Age has been strongly associated with obstructive CAD, which is consistent with most studies.^20^ Lappé et al^14^ and our study showed that age was strongly associated with obstructive CAD in univariate and multivariate analyzes. Furthermore, the gender of men and some diseases such as diabetes and hypertension were also considered classic risk factors for coronary artery disease proven in the literature.^21^ This study revealed a new point that serum ALT level is a potential factor in predicting the presence of obstructive CAD through the support vector machine model (**Figure 2**). The cohort of 37085 Korean patients in the study by Yun et al^22^ showed that ALT was associated with increased cardiovascular disease. The association between ALT and cardiovascular disease is also different between regions, with a positive association of ALT with CVD events in Asian populations and possible negative associations in North American and European populations.^23^ Furthermore, Liu et al^24^ concluded that a higher ALT could protect against coronary artery disease /myocardial infarction.

 Heart valve surgery teams need to comprehensively assess patients based on the personal risk factors to choose the best approach to evaluate coronary artery disease. Our research provided an updated perspective for clinicians about the frequency of obstructive CAD in patients undergoing heart valve surgery. Unlike previous results, this study revealed that the prevalence of CAD was relatively high in patients with valvular heart disease requiring surgery now in developing countries. So, it improved pre-operative and long-term outcomes for these patients. Our research provided an updated perspective for clinicians about the frequency of obstructive CAD in patients undergoing heart valve surgery. Unlike previous results, this study revealed that the prevalence of CAD was relatively high in patients with valvular heart disease requiring surgery now in developing countries. Heart valve surgery teams need to comprehensively assess patients based on the personal risk factors to choose the best approach to evaluate the presence of coronary artery disease. So, it improved pre-operative and long-term outcomes for these patients.

 Our study had some limitations. First, this study was a single-center study with limited sample size. Moreover, larger population-based and multicenter studies are needed to validate appropriate risk models. Secondly, we were unable to classify the underlying cause of the valve injury. Third, potential limitations could arise that comorbidity based on ICD-10 and a history of cardiovascular events may be downright concise.

## Conclusion

 The study revealed that approximately one-fifth of the population had obstructive coronary artery disease. The support vector machine model could better classify patients with obstructive and non-obstructive coronary artery disease. Furthermore, the essential variables that improved our model were typical angina, diabetes, hypertension, and age.

## Acknowledgements

 The authors are thankful to the Cardiology Department staff, Cho Ray Hospital.

## Competing Interests

 The authors declare that there is no potential conflict of interest regarding the publication of this article.

## Ethical Approval

 The study was carried out in accordance with the Declaration of Helsinki and approved by the ethics committee in the biomedical research of the University of Medicine and Pharmacy at Ho Chi Minh City,and Cho Ray Hospital (ID: 606/DHYD-HD).

## Funding

 No funding was used in this study
